# Selective Internal Radiation Therapy in Breast Cancer Liver Metastases: Outcome Assessment Applying a Prognostic Score

**DOI:** 10.3390/cancers13153777

**Published:** 2021-07-27

**Authors:** Imke Schatka, Monique Tschernig, Julian M. M. Rogasch, Stephanie Bluemel, Josefine Graef, Christian Furth, Jalid Sehouli, Jens-Uwe Blohmer, Bernhard Gebauer, Uli Fehrenbach, Holger Amthauer

**Affiliations:** 1 Department of Nuclear Medicine, Charité–Universitätsmedizin Berlin, Corporate Member of Freie Universität Berlin, Humboldt-Universität zu Berlin and Berlin Institute of Health, 13353 Berlin, Germany; Monique.tschernig@charite.de (M.T.); Julian.rogasch@charite.de (J.M.M.R.); Stephanie.bluemel@charite.de (S.B.); Josefine.graef@charite.de (J.G.); christian.furth@charite.de (C.F.); holger.amthauer@charite.de (H.A.); 2 Berlin Institute of Health (BIH), 10178 Berlin, Germany; 3 Department of Gynecology and Breast Center, Charité–Universitätsmedizin Berlin, Corporate Member of Freie Universität Berlin, Humboldt-Universität zu Berlin and Berlin Institute of Health, 13353 Berlin, Germany; jalid.sehouli@charite.de (J.S.); jens.blohmer@charite.de (J.-U.B.); 4 Department of Radiology, Charité–Universitätsmedizin Berlin, Corporate Member of Freie Universität Berlin, Humboldt-Universität zu Berlin and Berlin Institute of Health, 13353 Berlin, Germany; Bernhard.gebauer@charite.de (B.G.); uli.fehrenbach@charite.de (U.F.)

**Keywords:** radioembolization, Y-90 microspheres, breast cancer, liver metastases, prognostic score, overall survival

## Abstract

**Simple Summary:**

Selective internal radiation therapy (SIRT) is a treatment option for patients with breast cancer and liver metastases. However, there are currently no established factors to decide whether a certain patient will likely benefit from this treatment. This study analyzed the overall survival (the time from treatment to the patient’s death) in 38 patients with a total of 42 radioembolization procedures. Among all investigated factors, two variables were able to predict the outcome after radioembolization: The clinical performance status (ECOG) and the presence of elevated laboratory parameters that are markers of the liver damage (ALT, AST) before the start of the treatment. If none of these two risk factors was present, patients showed favorable outcome (average overall survival of 19.2 months). If both factors were present, overall survival after treatment was unfavorable (average of 2.2 months). In the future, this risk adapted prognostic score might help to elucidate which individual patient benefits from radioembolization.

**Abstract:**

Selective internal radiation therapy (SIRT) is a therapy option in patients with breast cancer liver metastasis (BCLM). This analysis aimed at identifying a prognostic score regarding overall survival (OS) after SIRT using routine pretherapeutic parameters. Retrospective analysis of 38 patients (age, 59 (39–84) years) with BCLM and 42 SIRT procedures. Cox regression for OS included clinical factors (age, ECOG and prior treatments), laboratory parameters, hepatic tumor load and dose reduction due to hepatopulmonary shunt. Elevated baseline ALT and/or AST was present if CTCAE grade ≥ 2 was fulfilled (>3 times the upper limit of normal). Median OS after SIRT was 6.4 months. In univariable Cox, ECOG ≥ 1 (hazard ratio (HR), 3.8), presence of elevated baseline ALT/AST (HR, 3.8), prior liver surgery (HR, 10.2), and dose reduction of 40% (HR, 8.1) predicted shorter OS (each *p* < 0.05). Multivariable Cox confirmed ECOG ≥ 1 (HR, 2.34; *p* = 0.012) and elevated baseline ALT/AST (HR, 4.16; *p* < 0.001). Combining both factors, median OS decreased from 19.2 months (0 risk factors; *n* = 14 procedures) to 5.9 months (1 factor; *n* = 20) or 2.2 months (2 factors; *n* = 8; *p* < 0.001). The proposed score may facilitate pretherapeutic identification of patients with unfavorable OS after SIRT. This may help to balance potential life prolongation with the hazards of invasive treatment and hospitalization.

## 1. Introduction

Breast cancer continues to be a leading cause of mortality worldwide, with approximately one out of two patients developing metastases over the course of the disease [1]. Patients with advanced disease develop hepatic metastases in up to 50%. In 5% to 12% of patients, liver metastases occur as the primary site of breast cancer recurrence [2]. Despite the high frequency and heterogeneity of breast cancer liver metastasis (BCLM) as documented in recent literature, BCLM is still accompanied by late diagnosis and poor outcomes [3].

Owing to the overall modest prognosis with median survival rates of less than eight months in untreated tumor lesions, there is an unmet need for alternative treatment modalities, combining a minimally invasive approach with a low morbidity and mortality rate [3]. Selective internal radionuclide therapy (SIRT) or else transarterial radioembolization (RE) with yttrium-90 (Y-90) labeled microspheres has been advocated as an option for such patients as it potentially inherits selective high-dose radiation to the target lesion and minimizes collateral damage to the normal liver parenchyma [4].

Although SIRT is generally well tolerated, relevant and potentially life-threatening side effects, which predominantly occur in patients after antineoplastic chemotherapy, have been reported. Radioembolization-induced liver disease (REILD) can appear in up to one out of three patients undergoing SIRT, ranging from clinically asymptomatic increase in liver transaminase blood levels up to hepatic failure [5]. In contrast, response rates and overall survival (OS) vary, ranging from 26% to 63% and 6 to 13.9 months, respectively [6,7,8,9,10]. Therefore, SIRT requires thorough preparation including pretherapeutic angiography to identify aberrant vessels or excessive hepatopulmonary shunts and the evaluation of remaining hepatic function [11].

Consequently, there is a high clinical need to identify parameters that are consistently and reliably associated with (un-)favorable outcome after SIRT to identify patients who are expected to benefit from the procedure. Previous studies regarding SIRT in BCLM have each identified different prognostic factors associated with OS, either the Eastern Cooperative Oncology Group (ECOG) performance status, the pattern and burden of hepatic tumor, baseline bilirubin or gamma-glutamyltransferase (GGT) levels [6,7,8,9,10]. However, except for a tumor load >25% highlighted by two studies [6,8], none of these parameters has been confirmed, and the clinical implications remain unclear.

Therefore, the aim of the present analysis was to investigate the value of the above-named prognostic parameters for OS in an independent patient cohort. The goal was to implement a simple prognostic score that could be used for the interdisciplinary assessment of the expected benefits in patients with BCLM who are potential candidates for SIRT.

## 2. Results

### 2.1. Patient Cohort

Of the 41 patients which met the inclusion criteria, three patients were lost to follow-up and subsequently excluded from further analysis.

A total of 38 patients were included into final analysis (median age, 59 years; interquartile range (IQR), 51 to 67 years; range, 39 to 84 years). Overall, 42 SIRT procedures were performed (Table 1), including 24 whole liver/single session treatments, 4 whole liver/sequential treatments (=8 procedures), 7 right liver lobe and 3 left liver lobe treatments. In the latter 10 patients, reasons for discontinuation of the sequential SIRT approach after the first procedure were either a stable disease in the contralateral liver lobe or unifocal disease that could be treated with local ablative treatment (*n* = 3), progressive extrahepatic disease (*n* = 3), insufficient liver function (*n* = 2) or inadequate anatomy of the hepatic blood vessels (*n* = 2), respectively.

The median tumor load relative to the volume of the treated liver lobe (or relative to the total liver volume if it was treated in a single session) was 7.9% (IQR, 3.4% to 18.2%; range, 0.5% to 78%; Table 2). Extrahepatic metastases at the time of SIRT were present in 27 of 42 SIRT procedures (64%). Liver metastases were deemed predominant by interdisciplinary consensus in all cases.

Extrahepatic metastases were present in 27 of 42 procedures (64%). This included bone metastases in 18 procedures (43%), lymph node metastases (mostly axillary) in 9 procedures (21%), pulmonary metastases in 2 (5%) and brain metastases in 3 procedures (7%). Bone metastases were often small and uni- or oligofocal (intervention required in 2 patients). Pulmonary metastases were small (<8 mm). Brain metastases were controlled by previous radiation therapy.

### 2.2. Treatment and Toxicities

One patient died by a cardiovascular event, nine days after SIRT, qualifying as a treatment-related death. Another patient required cardiopulmonary resuscitation during SIRT and survived. Further therapy-related complications included moderate gastritis in three (8%), nausea in three (8%) and vascular spasm in four (10%) patients. Four patients developed ascites (10%), while jaundice was noted in one patient over the post-therapeutic course.

In a third of patients, the prescribed SIRT activity was reduced due to an excessive hepatopulmonary shunt. More specifically, dose reduction by 20% or by 40% was required in 11 (26%) or three of 42 SIRT procedures (7%), respectively. A median activity of 1.42 GBq per SIRT procedure was administered (IQR, 1.0 to 1.9 GBq; range, 0.7 to 2.28 GBq).

### 2.3. Univariable Cox Regression

All patients deceased within the observation period. Median overall survival was 6.4 months (95% confidence interval (95% CI), 4.9 to 7.9 months; range, 0.3 to 37.4 months).

In univariable cox regression, higher ECOG performance status, the presence of any baseline transaminase elevation with CTCAE grade ≥ 2, prior surgical treatment, and a dose reduction for SIRT were each significantly associated with shorter OS (each *p* < 0.05; Table 3).

### 2.4. Multivariable Cox Regression

**Figure 1:**
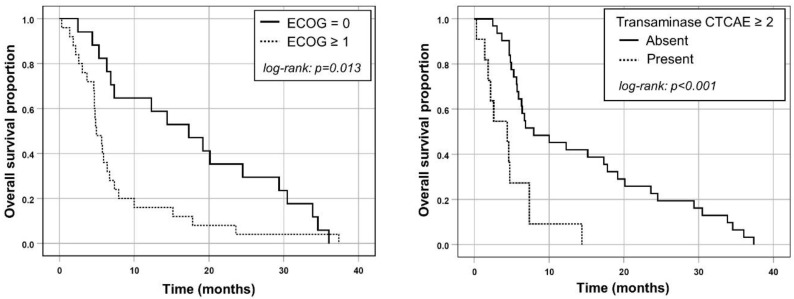
Kaplan–Meier plots for OS in patients separated either by their ECOG performance status or by the absence/presence of any transaminase elevation at baseline with CTCAE grade ≥ 2.

Multivariable Cox regression included ECOG ≥ 1, the presence of any elevated baseline liver transaminase with CTCAE grade ≥ 2, prior liver surgery and a dose reduction of 40% (Table 4). Both ECOG ≥ 1 (HR, 2.3; 95% CI, 1.2 to 4.5; *p* = 0.012) and the presence of any baseline transaminase with CTCAE grade ≥ 2 (HR, 4.2; 95% CI, 1.9 to 9.2; *p* < 0.001) remained significant predictors of shorter OS (Figure 1), while prior liver surgery and dose reduction were not significant (each *p* > 0.1).

### 2.5. Combined Prognostic Score

**Figure 2:**
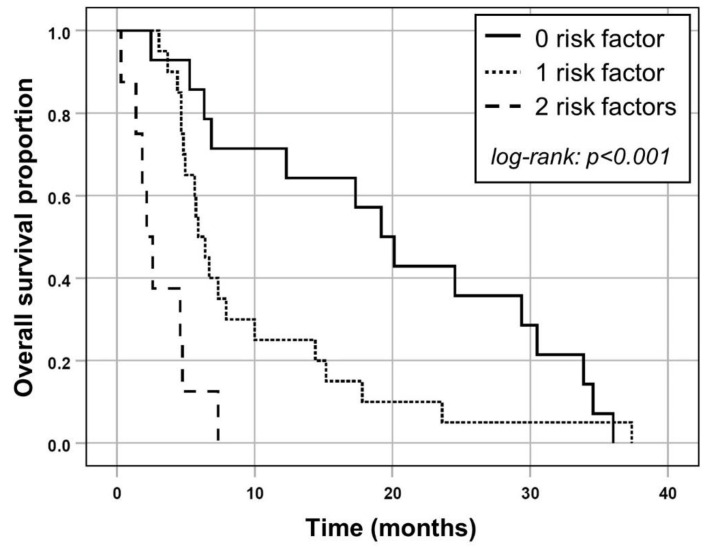
Kaplan–Meier plots for OS in patients separated by the number of risk factors according to the prognostic score based on ECOG ≥ 1 and/or the presence of any elevated liver transaminase at baseline (CTCAE grade ≥ 2).

Based on the results of multivariable Cox regression, ECOG ≥ 1 and the presence of any elevated baseline transaminase (ALT and/or AST) according to CTCAE grade ≥ 2 were included in a prognostic clinical score. In 8/42 SIRT procedures and both risk factors (19%), median OS was shorter (2.2 months; 95% CI, 1.1 to 3.2 months) compared to 20/42 procedures (48%) with one risk factor (median OS, 5.9 months; 95% CI, 4.5 to 7.3 months) or 14/42 SIRT treatments (33%) with none of both risk factors, respectively (median OS, 19.2 months; 95% CI, 14.0 to 24.4 months; log-rank test, *p* < 0.001; Figure 2).

## 3. Discussion

Breast cancer is the leading cause of cancer-related deaths in women. Liver metastases develop in half of patients with metastatic breast cancer and are a frequent cause of mortality among this subset of patients. As the clinical heterogeneity aggravates the ability to draw a firm conclusion on eligibility for SIRT, ensuring proper patient selection remains of utmost importance. To the best of our knowledge, no adequate prognostic score has been proposed for SIRT with Y-90 microspheres in BCLM to date, and results on prognostic factors from previous studies are mostly unconfirmed. The current study confirmed the previously reported prognostic value of ECOG performance status [9] and elevated baseline ALT/AST [7]. Both factors formed the proposed simple prognostic score which may help to identify patients with expected (un-)favorable outcome after SIRT in clinical routine.

More specifically, there is a growing body of evidence that impaired liver function of almost any degree is associated with a poor outcome [2,9]. In the current analysis, the elevated baseline ALT and/or AST were independent predictors of shorter OS after SIRT and the strongest predictor in multivariable Cox regression (HR, 4.16). This is in line with a previous report by Fendler et al. [7] who also investigated prognostic factors of OS after SIRT in 81 patients with BCLM. As in the present analysis, the authors showed that baseline transaminase toxicity of CTCAE grade ≥ 2 predicted shorter OS in multivariable Cox regression (HR, 2.15). The authors also demonstrated that a high liver tumor load ≥50% was independently associated with impaired OS (HR, 5.67). However, this could not be confirmed in the present study (Table 3). This may be explained by the lower percentage of patients with moderate (25–50%) or high tumor load (≥50%) in the current analysis (current study: 12% and <10%; previous study: 30% and 10%). It may also be explained by the observation that a higher number of current patients only underwent SIRT treatment of one liver lobe (10/42 = 24%) compared to Fendler et al. (8/81 = 10%) [7]. Furthermore, bilobar BCLM were not associated with worse OS than unilobar disease in the present analysis, which may be explained by the low number cases with unilobar metastases (1/42 procedures).

There is considerable controversy whether ECOG performance status is a consistent predictor of impaired OS. While Saxena et al. and Gordon et al. failed to find a significant association between ECOG performance status and OS, Pieper et al. as well as the results of the current study indicate that ECOG ≥ 1 confers a significantly worse prognosis [8,9,10].

Baseline bilirubin levels [8], GGT levels [9] and the pattern of disease (solitary vs. multifocal) [8] have been demonstrated as prognostic factors in patients with BCLM and SIRT in single studies. However, these results could not be confirmed in the current analysis. The small sample sizes and different investigated factors in each of the published single-center analyses implies that a pooled multicenter analysis of all available retrospective data may help to achieve the anticipated level of statistical power and validation. The observation that extrahepatic metastases were not associated with worse OS in the current patients is in line with Fendler et al. [7]. Notably, the frequency of patients with extrahepatic sites was similar between both studies (current study: 64% and Fendler et al.: 67%). Neither the presence of any extrahepatic metastases nor the number of metastatic sites or a weighted summed score of these sites was prognostic for OS in the present sample. This underlines that these patients had liver-dominant metastases which were deemed potentially life-limiting.

Median survival in women exhibiting liver metastases of breast cancer has been estimated to be 4–21 months, which compares unfavorably with metastases at other sites [12,13,14]. Even though Y-90 SIRT is advocated as a promising treatment option for unresectable chemo-resistant liver tumors, median survival rates remain less than desirable. The median OS in the present patient cohort of 6.4 months falls within the range previously reported in the literature. Gordon et al. observed a median OS of 6.6 months in 75 patients [8] whereas Pieper et al. reported a median OS of 6.0 months [9]. It should be noted, however, that markedly longer OS has also been documented, with Cianni et al. and Saxena et al. reporting a median OS of 11.5 months and 13.6 months, respectively [10,15]. Furthermore, Fendler et al. were able to demonstrate a median OS of 8.0 months in the largest cohort of 81 patients to date [7]. Notably, the presented prognostic score may help to identify patients who can be expected to show especially favorable OS after SIRT, as OS increased up to a median of 19.2 months if none of the two risk factors was present. If one risk factor was present, median OS was only slightly below the overall average (5.9 months). In contrast, OS was considerably shorter (2.2 months) in the presence of both risk factors. This may mean that patients with none or one of the unfavorable factors may continue to be candidates for SIRT treatment while in patients with both risk factors, the decision to recommend SIRT may require especially careful evaluation of the expected benefits.

The analysis of adverse events showed that SIRT in BCLM appears to be a relatively safe procedure. Similar to what has previously been reported in the relevant literature, the rate of patients developing clinical toxicity after treatment was low, typically classified as minor (CTCAE grade 1 or 2) and usually resolved without active intervention [8,16]. This may be partly attributed to a lower average dose of 1.42 GBq when compared to those reported in the literature ranging from 1.6 to 2.1 GBq [7,15,16]. The lower average treatment activity in the present study is likely due to the considerable number of single lobe SIRT procedures (24%) and one third of patients with dose reduction by 20% or 40% due to excessive hepatopulmonary shunt fraction.

The current analysis is limited by its retrospective nature and the inclusion of patients who only underwent SIRT of a single liver lobe according to interdisciplinary consensus recommendation. Due to the retrospective design, various serum markers, such as lactate dehydrogenase (LDH), which has been associated with worse prognosis in metastatic breast cancer [17,18], were not available in all patients and thus could not be delineated.

It needs to be acknowledged, that the current study lacks post-therapy dosimetry data. This is due to the retrospective patient enrollment and differences in post-therapy SPECT/CT protocols. In line with the results of Wyld et al. [19] patients mainly showed multiple small, non-confluent metastases of the liver, which complicated proper dosimetry via post-therapy SPECT/CT. However, all scans comprised of visual assessment of tracer accumulation in the liver metastases.

Even though image-based dosimetry is consistently investigated, its recommendations have yet to solidify a consensus on calculating image-based doses. In addition, there still is considerable controversy in current literature on the correlation between uptake in pre-treatment macroaggregated albumin imaging and therapy response. Garin et al. [20] demonstrated a good correlation, whereas Ulrich et al. [21] and Wondergem et al. [22] were unable to echo these results. This is why dosimetry via the MAA scans was not performed.

Above all else, the retrospective design may have resulted in distortion of adverse event reports arising from underreporting. However, patients were closely monitored, solely received the best supportive care after SIRT and the number of patients suffering from clinical toxicity fell into the expected range when compared to similar studies reported in the literature [23,24]. Due to a missing control group, the predictive value of the investigated variables could not be assessed. This would require a prospective, randomized controlled trial.

## 4. Materials and Methods

### 4.1. Study Design and Eligibility Criteria

This retrospective analysis was approved by the local ethics commission (Ethics committee of Charité–Universitaetsmedizin Berlin, approval number: EA1/288/16, approved on 25 October 2016) and carried out in accordance with the Tenets of the Declaration of Helsinki. The institutional database was retrospectively reviewed to identify patients with BCLM who underwent SIRT with Y-90 microspheres (vendor: SIRTEX Medical Pty. Ltd., St Leonards, NSW, Australia) between 2008 and 2015. According to in-house protocol, which is based on current guidelines [11], SIRT was performed based on interdisciplinary consensus in patients with BCLM, >18 years of age and preserved liver function (defined as bilirubin < 2 (mg/dL)) in a salvage situation (either refractory to all accepted therapy regimen at the time of admission or refusal of or non-eligibility to further systemic therapies after at least one cycle). After SIRT all patients needed to be treated by best supportive care.

Patient records were reviewed for baseline clinical data including the Eastern Cooperative Oncology Group performance status (ECOG). The histological subtype of breast cancer, the initial hormonal receptor status and human epidermal growth factor receptor 2 (HER2/neu) receptor as well as prior liver-directed or systemic treatments were documented. Baseline laboratory markers included the total bilirubin level, serum transaminase levels (alanine transaminase (ALT); aspartate transaminase (AST)) and GGT.

The elevated baseline ALT and/or AST were defined as any of the two transaminases elevated to >3 times the upper limit of normal (ULN). This corresponds to ALT > 93 U/L and/or AST > 105 U/L, respectively. This definition would be identical to Fendler et al. [7].

The volume of the whole liver and—in case of unilobar SIRT—the volume of the treated liver lobe were derived from pretherapeutic gadolinium-enhanced magnetic resonance imaging (MRI) of the liver using Eclipse (Varian Medical System, Palo Alto, CA, USA). This included manual delineation of the hepatic tumor volume in each liver lobe. In patients with contraindications for MRI, CT was performed. The extent of extrahepatic disease at the time of SIRT was assessed by either x-ray or staging CT of the thorax as well as MRI or CT of the abdomen. None of the patients had been diagnosed with liver cirrhosis at the time of SIRT. However, as part of this analysis the presence of radiological signs of liver cirrhosis was reviewed using either gadolinium-enhanced MRI (*n* = 39 SIRT procedures) or contrast-enhanced CT of the liver (*n* = 3).

Finally, for the applied SIRT activity, any reduction in the prescribed activity by 20% or 40% due to increased hepatopulmonary shunt, the extent of SIRT (whole liver vs. unilobar), and the number of SIRT sessions were reviewed.

### 4.2. Radioembolization

SIRT was performed in accordance with the guidelines of the Radioembolization Brachytherapy Oncology Consortium (REBOC), and as described in detail elsewhere [11,25]. The activity of Y-90 microspheres was calculated according to the modified body surface area (mBSA) method, where activity is reduced relative to the BSA method, based on lung shunt fraction and tumor involvement [26,27,28].

The relative hepatopulmonary shunt was evaluated from planar images of the pre-SIRT evaluation with TC-99m macroaggregated albumin. If the shunt was 10–15% or 15–20%, the prescribed SIRT activity was reduced by 20% or 40%, respectively [11]. If both liver lobes were treated, this was performed either in a single session or as a sequential protocol, depending on the estimated individual risk of posttherapeutic liver failure. In single-session as well as sequential procedures, the planned activity for each liver lobe was administered selectively into the left or right liver lobe, respectively.

### 4.3. Assessment of Toxicity and Survival

After SIRT, all patients were closely monitored as inpatients; this included daily clinical examination as well as blood tests of liver function and coagulation after the SIRT procedure. Patients with symptoms of acute toxicity of SIRT were not discharged before substantial improvement was documented. All patients were routinely followed-up every three months at our department, which included thorough documentation of the clinical performance status, laboratory parameters as well as CT/MRI follow-up imaging.

REILD was defined as serum total bilirubin of ≥3.0 mg/dL and ascites of grade ≥ 2 according to the CTCAE within 12 weeks after radioembolization in the absence of tumor progression or bile duct obstruction.

Overall survival (OS) was defined as the interval between the date of SIRT until death of any cause.

### 4.4. Statistical Analysis

Statistical analysis was primarily performed using SPSS 22 (IBM Corporation, Armonk, NY, USA). Non-parametric data distribution was assumed based on the Shapiro–Wilk test, and descriptive parameters were expressed as median, interquartile range (IQR) and range. Univariable Cox proportional hazards regression was used to investigate the association of all baseline variables with OS. The liver tumor load was categorized into <25% vs. 25–50% vs. ≥50% tumor load according to Fendler et al. [7]. The number of sites of extrahepatic metastases was calculated and included as a metric variable. To account for the potentially different influence of different metastatic sites on patient OS, a summed score was calculated with different weights for the presence of specific metastatic sites (lymph node metastases: 1 point; bone metastases: 2; pulmonary metastases: 2; brain metastases: 4 points). For instance, this summed score in a patient with lymph node and bone metastases would be 3. Variables with *p* ≤ 0.05 in univariable Cox regression were included in a multivariable Cox analysis. It was ensured that these variables did not violate the proportional hazards assumption using the goodness-of-fit test (function cox.zph) in the survival package of R (The R Project for Statistical Computing, v4.0.2). Variables that remained independent predictors of OS in multivariable analysis were included in a prognostic model. The survival probabilities of the patient groups in relation to the (binarized) individual variables and the combined score were estimated using the Kaplan–Meier method with the log-rank test and illustrated using Kaplan–Meier curves. Statistical significance was assumed at α = 0.05.

## 5. Conclusions

This analysis is in line with previous results, confirming the prognostic value of ECOG ≥ 1 and the presence of elevated baseline ALT/AST regarding OS after SIRT in patients with BCLM. The proposed simple prognostic score may help balancing the desired prolonged OS with the potential hazards of aggressive treatment protocols, and may consequently facilitate a targeted pretherapeutic patient selection. Further studies are warranted to validate these results prospectively with special considerations of subsequent therapies and patients related outcomes (PROMS).

## Figures and Tables

**Table 1:** Patient characteristics. All percentages are based on the number of SIRT procedures (*n* = 42).

Variables	Number (%) or Median (IQR; Range)
SIRT procedures	42 (100)
Age (years)	59 (IQR, 51–67; range, 39–84)
Female sex	42 (100)
ECOG performance status	
0	17 (40)
1	14 (33)
2	11 (26)
Baseline laboratory parameters	
Total bilirubin (mg/dL)	0.47 (IQR, 0.3–0.69; range, 0.2–1.16)
ALT (U/L)	48 (IQR, 30–76; range, 14–214)
AST (U/L)	65 (IQR, 38–102; range, 19–196)
GGT (U/L)	163 (IQR, 78–487; range, 24–2041)
Histological type (*n* = 5 missing)	
ductal breast cancer	30 (71)
lobular breast cancer	5 (12)
papillary breast cancer	2 (5)
Pattern of liver metastases	
unilobar	1 (2)
bilobar	41 (98)
Extrahepatic metastases	27 (64)
one metastatic site	22 (54)
two metastatic sites	5 (12)
summed, weighted score of metastatic sites	2 (IQR, 0–2; range, 0–6)
Radiological signs of liver cirrhosis	7 (17%)
Hormone receptor status	
estrogen receptor positive (*n* = 1 missing)	34 (83)
progesterone receptor pos. (*n* = 2 missing)	24 (60)
HER2/neu positive (*n* = 3 missing)	12 (31)
Hepatopulmonary shunt (%)	6.8 (IQR, 5.5–11.2; range, 2.3–19.0)
Prior treatment	42 (100)
Chemotherapy	42 (100)
Antihormonal therapy	32 (76)
Local treatment	11 (26)
Liver surgery	2 (5)
SIRT	1 (2)

**Table 2:** Characteristics of baseline liver volumes and tumor volumes. In patients who did not undergo SIRT of the whole liver, the tumor load may differ between the total liver and the treated lobe. All percentages are based on the number of SIRT procedures (*n* = 42).

Baseline Tumor Load	Number (%) or Median (IQR; Range)
Total liver volume (mL)	1497 (IQR, 1248–1970; range, 1009–4563)
Total liver tumor volume (mL)	121 (IQR, 54–371; range, 8–3350)
Total liver tumor load (%)	7.8 (IQR, 3.6–18.2; range, 0.5–73)
<25%	35 (83)
25 to <50%	5 (12)
≥50%	2 (5)
Tumor load in the treated lobe(s) (%)	7.9 (IQR, 3.4–18.2; range, 0.5–78)
<25%	34 (81)
25 to <50%	5 (12)
≥50%	3 (7)

**Table 3:** Univariable Cox regression. Significant results are printed in bold.

Variable	HR (95% CI)	*p*-Value
Age (years)	1.01 (0.98–1.04)	0.56
ECOG performance status		0.005
0	reference	
1	3.8 (1.69–8.54)	0.001
2	1.55 (0.7–3.43)	0.28
ECOG ≥ 1	2.24 (1.17–4.28)	0.015
Baseline laboratory parameters		
Total bilirubin (mg/dL)	1.33 (0.34–5.17)	0.68
Any transaminase elevation of CTCAE grade ≥ 2	3.78 (1.76–8.13)	0.001
GGT (U/L)	1.001 (1.0–1.002)	0.16
Pattern of liver metastases		
bilobar vs. unilobar	1.3 (0.2–9.7)	0.8
Extrahepatic metastases		
- Presence vs. absence	1.38 (0.73–2.64)	0.32
- Number of metastatic sites	1.36 (0.84–2.2)	0.22
- Summed, weighted score	1.15 (0.93–1.43)	0.21
Radiological signs of liver cirrhosis	1.31 (0.57–3.0)	0.52
Baseline tumor load		
Total liver tumor load (%)	0.97 (0.2–5.4)	0.97
<25%	reference	
25 to <50%	0.97 (0.37–2.56)	0.96
≥50%	0.75 (0.18–3.21)	0.7
Tumor load in the treated lobe(s) (%)	1.17 (0.19–7.18)	0.86
<25%	reference	
25 to <50%	0.74 (0.28–1.92)	0.53
≥50%	1.11 (0.33–3.75)	0.86
Prior treatment		
Antihormonal therapy	0.75 (0.36–1.55)	0.44
Local treatment	0.9 (0.43–1.87)	0.78
Liver surgery	10.2 (2.0–51.4)	0.005
Dose reduction (%)		0.012
0%	reference	
20%	0.9 (0.45–1.83)	0.78
40%	8.08 (1.97–33.05)	0.00
SIRT activity (GBq)	0.49 (0.24–1.01)	0.054
Hormone receptor status		
estrogen receptor positive (*n* = 1 missing)	0.65 (0.28–1.49)	0.31
progesterone receptor positive (*n* = 2 missing)	0.54 (0.27–1.05)	0.069
HER2/neu positive (*n* = 3 missing)	0.95 (0.47–1.89)	0.88

HR, hazard ratio; 95% CI, 95% confidence interval.

**Table 4:** Multivariable Cox regression. Significant results are printed in bold.

Variable	HR (95% CI)	*p*-Value
Baseline ECOG performance status ≥ 1	2.34 (1.2–4.54)	0.012
Any baseline transaminase with CTCAE grade ≥ 2	4.16 (1.87–9.23)	<0.001
Prior liver surgery	4.12 (0.55–31.0)	0.17
Dose reduction by 40%	3.57 (0.64–19.8)	0.15

HR, hazard ratio; 95% CI, 95% confidence interval.

## Data Availability

The data presented in this study are available on request from the corresponding author. The data are not publicly available due to data privacy.
